# Supplementary material for: Splice-Junction-Based Mapping of Alternative Isoforms in the Human Proteome
Source: Cell Rep. Author manuscript; Available in PMC 2020 Jan 15. (PMC6961840; doi:10.1016/j.celrep.2019.11.026)
Supplement: 2 [file NIHMS1546469-supplement-2.zip › DF1/PXD010154/Thyroid/Thyroid_35_TG_AVRSKVPDSEFPVMQCLTVAQNNAPSFCPLVVLPSLTEK.pdf]

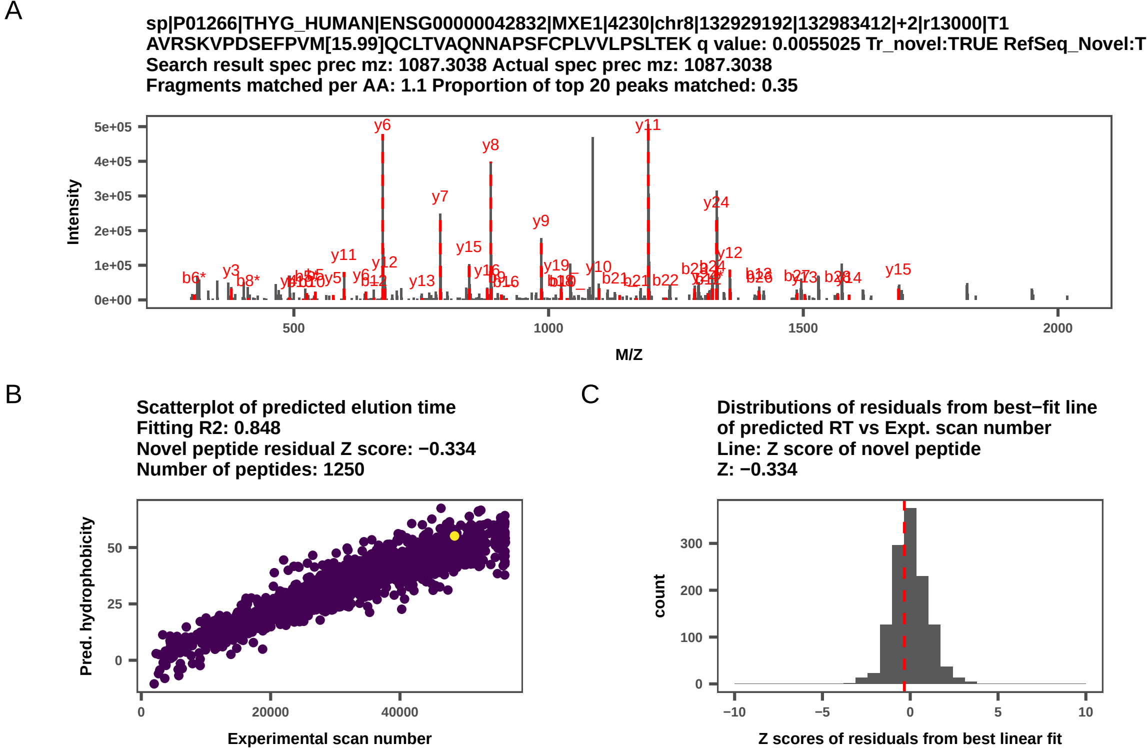
A
sp|P01266|THYG_HUMAN|ENSG00000042832|MXE1|4230|chr8|132929192|132983412|+2|r13000|T1
AVRSKVPDSEFPVM[15.99]QCLTVAQNNAPSFCPLVVLPSLTEK q value: 0.0055025 Tr_novel:TRUE RefSeq_Novel:T
Search result spec prec mz: 1087.3038 Actual spec prec mz: 1087.3038
Fragments matched per AA: 1.1 Proportion of top 20 peaks matched: 0.35
Intensity
5e+05
4e+05
3e+05
2e+05
1e+05
0e+00
y6
y8
y11
y7
y24
y9
y15
y12
y13
y10
b22
M/Z
500
1000
1500
2000
B
Scatterplot of predicted elution time
Fitting R2: 0.848
Novel peptide residual Z score: −0.334
Number of peptides: 1250
Pred. hydrophobicity
50
25
0
Experimental scan number
0
20000
40000
C
Distributions of residuals from best−fit line
of predicted RT vs Expt. scan number
Line: Z score of novel peptide
Z: −0.334
count
300
200
100
0
Z scores of residuals from best linear fit
−10
−5
0
5
10
